# Applications and limitations of Centers for Disease Control and Prevention miniature light traps for measuring biting densities of African malaria vector populations: a pooled-analysis of 13 comparisons with human landing catches

**DOI:** 10.1186/s12936-015-0761-9

**Published:** 2015-06-18

**Authors:** Olivier J T Briët, Bernadette J Huho, John E Gimnig, Nabie Bayoh, Aklilu Seyoum, Chadwick H Sikaala, Nicodem Govella, Diadier A Diallo, Salim Abdullah, Thomas A Smith, Gerry F Killeen

**Affiliations:** Department of Epidemiology and Public Health, Swiss Tropical and Public Health Institute, Socinstrasse 57, 4002 Basel, Switzerland; University of Basel, Petersplatz 1, Basel, 4003 Switzerland; Ifakara Health Institute, PO Box 78373, Dar es Salaam, United Republic of Tanzania; Centre for Global Health Research, Kenya Medical Research Institute, PO Box 1578, Kisumu, Kenya; Division of Parasitic Diseases, Centers for Disease Control and Prevention, Atlanta, 4770 Buford Highway, Mailstop F-42, Atlanta, GA 30341 USA; Centers for Disease Control and Prevention, PO Box 1578, Kisumu, Kenya; Vector Biology Department, Liverpool School of Tropical Medicine, Pembroke Place, Liverpool, L3 5QA UK; National Malaria Control Centre, Chainama Hospital College Grounds, Off Great East Road, PO Box 32509, Lusaka, Zambia; Centre National de Recherche et de Formation sur le Paludisme (CNRFP), 01 BP 2208, Ouagadougou 01, Ouagadougou, Burkina Faso

**Keywords:** Human landing catch, CDC light traps, *Anopheles gambiae*, *Anopheles funestus*

## Abstract

**Background:**

Measurement of densities of host-seeking malaria vectors is important for estimating levels of disease transmission, for appropriately allocating interventions, and for quantifying their impact. The gold standard for estimating mosquito—human contact rates is the human landing catch (HLC), where human volunteers catch mosquitoes that land on their exposed body parts. This approach necessitates exposure to potentially infectious mosquitoes, and is very labour intensive. There are several safer and less labour-intensive methods, with Centers for Disease Control light traps (LT) placed indoors near occupied bed nets being the most widely used.

**Methods:**

This paper presents analyses of 13 studies with paired mosquito collections of LT and HLC to evaluate these methods for their consistency in sampling indoor-feeding mosquitoes belonging to the two major taxa of malaria vectors across Africa, the *Anopheles gambiae* sensu lato complex and the *Anopheles funestus* s.l. group. Both overall and study-specific sampling efficiencies of LT compared with HLC were computed, and regression methods that allow for the substantial variations in mosquito counts made by either method were used to test whether the sampling efficacy varies with mosquito density.

**Results:**

Generally, LT were able to collect similar numbers of mosquitoes to the HLC indoors, although the relative sampling efficacy, measured by the ratio of LT:HLC varied considerably between studies. The overall best estimate for *An. gambiae* s.l. was 1.06 (95% credible interval: 0.68–1.64) and for *An. funestus* s.l. was 1.37 (0.70–2.68). Local calibration exercises are not reproducible, since only in a few studies did LT sample proportionally to HLC, and there was no geographical pattern or consistent trend with average density in the tendency for LT to either under- or over-sample.

**Conclusions:**

LT are a crude tool at best, but are relatively easy to deploy on a large scale. Spatial and temporal variation in mosquito densities and human malaria transmission exposure span several orders of magnitude, compared to which the inconsistencies of LT are relatively small. LT, therefore, remain an invaluable and safe alternative to HLC for measuring indoor malaria transmission exposure in Africa.

**Electronic supplementary material:**

The online version of this article (doi:10.1186/s12936-015-0761-9) contains supplementary material, which is available to authorized users.

## Background

Direct estimates of the rates at which humans are bitten by vector mosquitoes are invaluable indicators of humans’ risk of exposure to vector-borne pathogens. The numbers of host-seeking mosquitoes caught by trapping methods which catch the majority of vectors attacking surveyed hosts can be used to estimate the human biting rate (HBR), a basic parameter in assessing transmission of any mosquito-borne disease and malaria in particular [1]. The HBR, when multiplied by the prevalence of sporozoites in mosquitoes, gives an estimate of the entomological inoculation rate (EIR), a measure of human exposure to malaria transmission [2].

The human landing catch (HLC), in which human volunteers catch mosquitoes that land on their exposed body parts, is considered the ‘gold standard’ method for measuring exposure of humans to mosquito bites [3, 4]. However, this method is difficult to justify for most applications due to the deliberate exposure of humans to potentially infectious bites from vectors of human disease. While it is possible to protect HLC participants from *Plasmodium falciparum* with anti-malarial drug prophylaxis [5], the same cannot be said of most arboviruses for which preventative drugs and vaccines are not available. It is also uncomfortable, and labour-intensive to the point that it is impossible to sustain on a large scale.

Several other methods that do not require human exposure have been developed as alternatives to HLC for estimating the HBR [6]. For African malaria vectors, placing Centers for Disease Control Miniature Light Traps (LT) near human-occupied bed nets inside houses is by far the most widely used and evaluated method [7–11] but other alternatives include bed net traps [12–14], tent traps [15–19] and odour-baited traps [20].

In general, the numbers of host-seeking mosquitoes of a given taxon captured by different methods are strongly correlated but data from two methods can be highly correlated without the methods necessarily sampling from the same mosquito population [21]. If two trapping methods are sampling from the same mosquito population, albeit potentially with different trapping efficiencies, the numbers of mosquitoes they report should be proportionate over the whole range of mosquito densities. However, traps often capture zero mosquitoes during the night and mosquito densities vary over many orders of magnitude. This makes standard methods for assessing agreement between different measurement techniques [21, 22] inapplicable. The way in which the zero observations are handled can make important differences to inferences about proportionality in numbers caught [23].

The sampling efficacy for malaria vectors of LT as compared to HLC has been evaluated in several areas with diverse outcomes [8–10, 24–31]. Discrepancies between studies might be the result of differences in statistical approach, or other methodological differences, for instance in placement of the trap [32] or spatial and temporal variations in environmental conditions or the behaviour of local mosquito populations. The pooled analysis presented here is an attempt to overcome these ambiguities by analysing proportionality in numbers of mosquitoes caught and the relative trapping efficacy of LT, compared to HLC, using data from 13 separate studies from 12 diverse settings across five African countries, encompassing a wide range of vector densities.

## Methods

### Study sites

**Figure 1:**
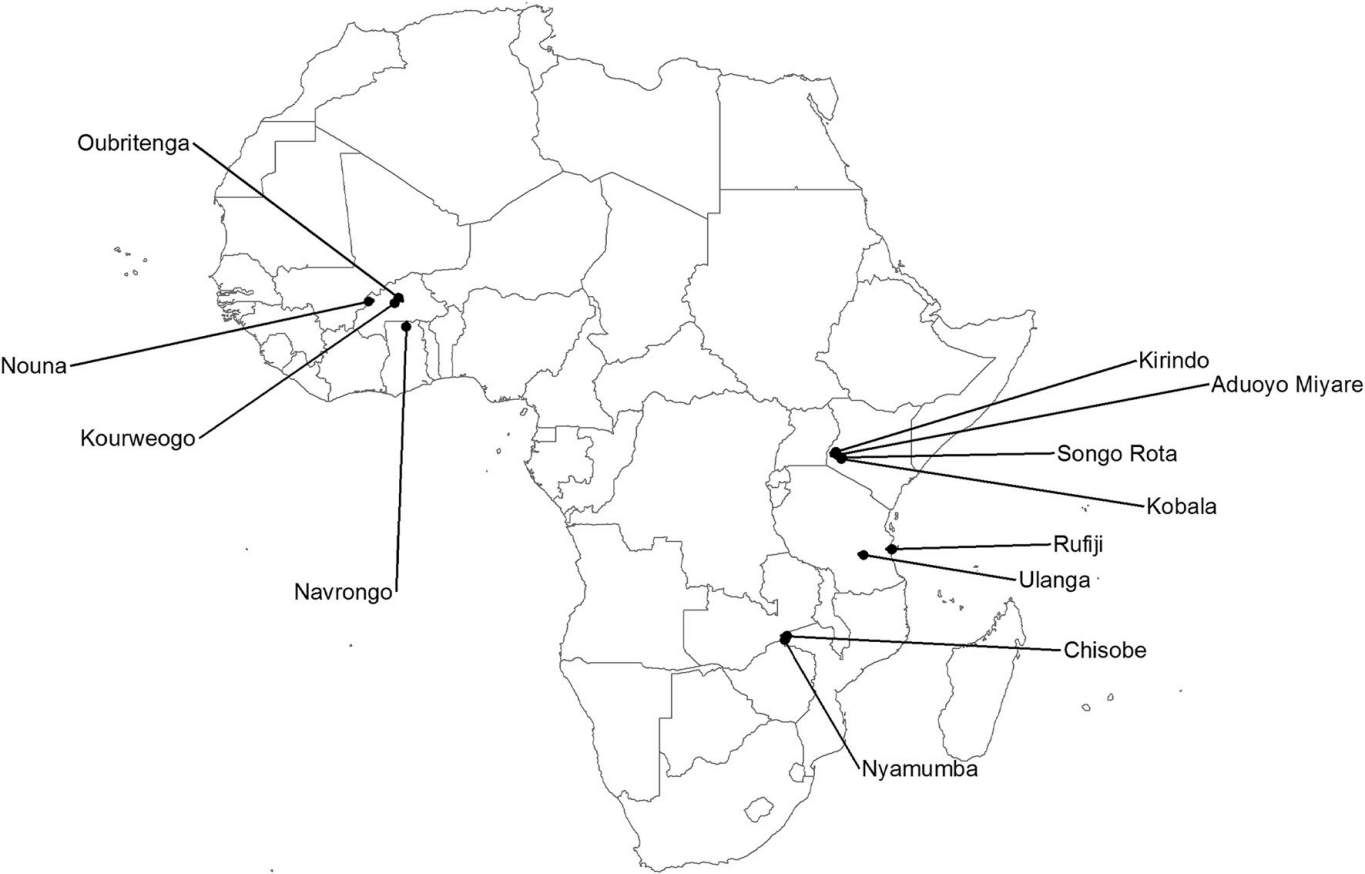
Location of study sites. Map of Africa showing the location of study sites where data for this analysis were collected.

A dataset allowing direct comparison of indoor LT to indoor HLC in multiple sites across rural Africa (Figure 1) was compiled, mainly from multi-country studies of malaria transmission and epidemiology: the Malaria Transmission Intensity and Mortality Burden Across Africa (MTIMBA) [33] and Malaria Transmission Consortium (MTC) [34] projects. The MTIMBA studies were conducted between 2001 and 2004, including studies in Burkina Faso (Oubritenga, Kourweogo and Nouna), Tanzania (Ulanga and Rufiji) and Ghana (Navrongo). The MTC studies were conducted between 2006 and 2010, at sites in Zambia (Chisoba and Nyamumba) and Kenya (Aduoyo Miyare, Songo Rota, Kirindo and Kobala) (Table 1). Additional data for validation were obtained from earlier studies in Tanzania and from Bioko, Equatorial Guinea [24].

**Table 1:** Study details

	Site	Country	Platform	Year(s)	Dominant species		References
					*An. gambiae* complex	*An. funestus* group	
1	Aduoyo Miyare	Kenya	MTC	2009	*arabiensis*	*funestus*	[19]
2	Chisobe	Zambia	MTC	2009–2010	*quadriannulatus*	*funestus*	[18]
3	Kirindo	Kenya	MTC	2009	*arabiensis*	*funestus*	[19]
4	Kobala	Kenya	MTC	2009	*arabiensis*	*funestus*	[19]
5	Kourweogo	Burkina Faso	MTIMBA	2001–2004	*gambiae*	*funestus*	[71]
6	Navrongo	Ghana	MTIMBA	2001–2004	*gambiae*	*funestus*	[62]
7	Nouna	Burkina Faso	MTIMBA	2001–2004	*gambiae*	*funestus*	[72]
8	Nyamumba	Zambia	MTC	2009–2010	*quadriannulatus*	*funestus*	[18]
9	Oubritenga	Burkina Faso	MTIMBA	2001–2004	*gambiae*	*funestus*	[71]
10	Rufiji	Tanzania	MTIMBA	2001–2004	*gambiae*	*funestus*	[73]
11	Sango Rota	Kenya	MTC	2009	*arabiensis*	*funestus*	[19]
12	Ulanga 2004	Tanzania	MTIMBA	2004	*gambiae*	*funestus*	[74]
13	Ulanga 2006	Tanzania	MTIMBA	2006	*gambiae*	*funestus*	[74]

*MTC* Malaria Transmission Consortium, *MTIMBA* Malaria Transmission Intensity and Mortality Burden across Africa.

### Light trap calibration studies

For each study, standardized mosquito sampling protocols were used [6, 19]. LT were hung beside a sleeping place where one human volunteer slept covered by a bed net, the treatment status of which was not controlled and apparently had little effect [31, 35]. The LT was hung at the foot of the sleeping place at about 1.5 m above the floor [32]. The volunteers switched the LT on before going to bed, while the mosquito collectors switched the traps off in the morning. HLCs were done by volunteers that sat indoors and outdoors collecting mosquitoes which landed on their exposed limbs, using torchlight and aspirators [3]. For most of the MTIMBA studies, two pairs of volunteers conducted the HLC at each sampling point, with one pair replacing the other after the sixth hour (e.g., at midnight if catches started at 18.00 h). Within a pair, the volunteers interchanged positions (indoors or outdoors) hourly. In the MTC studies [18, 19] and the MTIMBA studies at the Ulanga site, only one pair of volunteers conducted HLC throughout the night, indoors and outdoors, without exchanging positions. Within each hour, they collected mosquitoes for 45 min and rested for 15 min [36]. The mosquito counts were not adjusted for the resting breaks of the volunteers.

In most of the MTIMBA studies, data included up to 3 years of daily indoor LT collections together with occasional HLC collections carried out for 48 nights of trapping (i.e., 24 periods of two consecutive nights of collection) spread over a year. The standard procedure involved classification of the human population into geographical clusters of about 100 people who were living in the same area, based on each study’s demographic database. Each month, at least 30 clusters (defined as groups of people geographically centred on index persons) were selected by simple random sampling from the demographic database. Each of the selected clusters was visited in a logistically convenient order each month. Each month, the index person and three additional people recruited from a randomized list of households in the same cluster were assigned LT for each collection night. The nearest compound to the index person was selected for indoor and outdoor HLC [15, 18, 19, 37]. Collection intensity and duration varied among studies. In the Ulanga study, an initial MTIMBA study in 2004 and two distinct additional studies, separated only by a few days at the end of 2006 in the same village, were all conducted using essentially identical methodology.

### Protection of human subjects and ethical approval

Ethical clearance was obtained from respective local ethical review bodies. Participants were educated on the study procedures and were made aware of the health risks involved by their participation. As precautionary measure, study participants were screened regularly for malaria infection by microscopy or rapid diagnostic test, followed by treatment of positive cases as per the local malaria treatment guidelines. In MTC studies, volunteers were given malaria prophylaxis, specifically Lariam^®^ (mefloquine) was provided in Kenya, Malarone^®^ (atovaquone and proguanil hydrochloride) in Tanzania and Deltaprim^®^ (dapsone and pyrimethamine) in Zambia.

### Data analysis

Agreement between the two methods was analysed by an extension of the method initially described previously [27]. Only trap counts for mosquitoes identified as members of the *Anopheles gambiae* complex and *Anopheles funestus* group that include the most important primary vectors in Africa, were included in the analysis. For each of these two important taxa, only strata (collections by two methods matched by location and night) where at least one mosquito was captured by one of the sampling methods (indoor HLC and LT) were included in the analysis. Data on outdoor HLC were excluded from the analysis as these are, in principle, not directly comparable with indoor collections by either HLC or LT. The number of strata included in the analysis varied by mosquito species taxon and study (Table 2). Those studies where less than ten strata were available per vector taxon were excluded from the analysis (Table 2).

**Table 2:** Data used in the modelling and model results

Site/species	Number of traps nights		Number of mosquitoes		Model 1	Model 2	
	LT	HLC	LT	HLC	α_s_	α_s_	γ_s_
*An. gambiae* s.l.							
Aduoyo Miyare	31	31	141	181	0.79 (0.63, 0.98)	0.36 (0.15, 0.63)	1.80 (1.29, 2.61)
Chisobe	44	44	507	275	1.83 (1.58, 2.12)	22.3 (12.3, 37.0)	0.55 (0.45, 0.68)
Kirindo	28	28	162	71	2.2 (1.69, 2.89)	0.57 (0.27, 0.89)	3.80 (2.16, 7.02)
Kobala	17	18	18	25	0.84 (0.48, 1.43)	0.57 (0.26, 0.95)	2.09 (1.19, 4.43)
Kourweogo	78	81	662	637	1.06 (0.95, 1.18)	0.40 (0.27, 0.56)	1.58 (1.38, 1.81)
Navrongo	76	76	3,316	3,865	0.86 (0.82, 0.90)	0.26 (0.19, 0.35)	1.39 (1.29, 1.48)
Nouna	78	80	1,834	812	2.39 (2.20, 2.60)	0.37 (0.29, 0.48)	1.58 (1.49, 1.67)
Nyamumba	43	43	277	130	2.09 (1.71, 2.57)	4.66 (2.51, 7.47)	0.75 (0.57, 1.01)
Oubritenga	108	113	771	791	0.97 (0.88, 1.08)	0.66 (0.49, 0.88)	1.18 (1.06, 1.32)
Rufiji	6	6	27	24		NA	NA
Sango Rota	32	32	31	87	0.41 (0.27, 0.60)	0.40 (0.16, 0.87)	0.62 (0.34, 0.99)
Ulanga 2004	22	44	1,005	6,403	0.33 (0.31, 0.36)	1.22 (0.41, 2.72)	0.75 (0.63, 0.91)
Ulanga 2006	36	18	4,008	1,477	1.36 (1.28, 1.44)	0.06 (0.03, 0.11)	2.92 (2.52, 3.40)
*An. funestus* s.l.							
Aduoyo Miyare	29	29	41	45	0.97 (0.65, 1.44)	0.69 (0.40, 0.96)	5.98 (1.84, 17.5)
Chisobe	52	52	1,692	1,101	1.53 (1.43, 1.66)	9.8 (5.95, 15.56)	0.69 (0.6, 0.78)
Kirindo	5	5	1	4	NA	NA	NA
Kobala	3	3	4	1	NA	NA	NA
Kourweogo	34	33	23	14	1.68 (0.97, 2.99)	1.48 (0.89, 3.84)	2.06 (0.84, 5.41)
Navrongo	75	75	4,373	2,018	2.19 (2.08, 2.30)	0.06 (0.04, 0.08)	5.56 (5.03, 6.13)
Nouna	57	56	819	267	3.20 (2.78, 3.69)	0.42 (0.29, 0.57)	1.96 (1.76, 2.20)
Nyamumba	55	55	938	648	1.45 (1.31, 1.60)	4.33 (2.68, 6.59)	0.75 (0.65, 0.87)
Oubritenga	62	61	41	56	0.83 (0.55, 1.22)	0.72 (0.41, 1.05)	1.42 (0.62, 4.75)
Rufiji	5	5	37	1	NA	NA	NA
Sango Rota	1	1	0	1	NA	NA	NA
Ulanga 2004	22	40	41	70	1.13 (0.79, 1.61)	0.63 (0.35, 0.97)	5.49 (1.46, 13.3)
Ulanga 2006	22	11	24	4	2.20 (1.11, 5.21)	1.45 (0.56, 3.02)	3.25 (0.98, 18.1)

Numbers between parentheses are 95% credible intervals.

$\alpha_{s}$ site specific sampling efficacy, $\gamma_{s}$ exponent testing proportionality, *NA* not analysed because less than ten trap nights of data were available.

In order to estimate the sampling efficiencies of LT relative to HLC, the following statistical model was used:

1 $$E\left( {y_{i} } \right) = \alpha_{s} E\left( {x_{i} } \right)$$

where $E\left( {y_{i} } \right)$ is the expected number of mosquitoes of a given taxon caught using LT in stratum *i*; $E\left( {x_{i} } \right)$ is the expected number of mosquitoes of the same taxon caught using the human landing method in the same stratum *i*; $\alpha_{s}$ is the relative sampling efficacy corresponding to study *s*, compared to HLC for which the value is set to unity. The underlying mosquito density $E\left( {x_{i} } \right)$ is assumed to have a log-normal distribution, i.e., $\ln \left( {E\left( {x_{i} } \right)} \right)\sim Normal\left( {\mu_{s} ,\sigma_{s}^{2} } \right)$, Poisson errors were assumed in the observed numbers of mosquitoes caught by any of the two methods so that: $x_{i} \sim Poisson\left( {E\left( {x_{i} } \right)} \right)$ and: $y_{i} \sim Poisson\left( {E\left( {y_{i} } \right)} \right)$ and the model therefore assumes the distribution of the numbers of mosquitoes caught by any method to be a log-normal mixture of Poisson distributions, and that HLC gives an unbiased estimate of the true mosquito density.

To allow for stochastic variation between studies, and to obtain an estimate of the overall average sampling efficacy across studies, the logarithms of the study-specific sampling efficacy, $\ln (\alpha_{s} )$, were assumed to vary normally about the overall average of the species complex, $\ln (\tilde{\alpha }_{t} )$, i.e.: $\ln (\alpha_{s} )\sim Normal\left( {\ln (\tilde{\alpha }_{t} ),\tilde{\sigma }_{t}^{2} } \right)$, thus leading to a hierarchical statistical model which was fitted using a Bayesian Markov chain Monte Carlo algorithm in the software WinBUGS version 1.4 [38]. The parameters $\alpha_{s}$, $\mu_{s}$, $\sigma_{s}$, $\tilde{\alpha }_{t}$, and $\tilde{\sigma }_{t}$ were assigned weakly informative prior distributions which constrained them to be positive but otherwise did not impose important constraints. The choice of prior between several different distributional forms was found to make negligible difference to the results.

To examine whether the sampling efficacy varied with the average mosquito density, the following extended model was also fitted:

2 $$E\left( {y_{i} } \right) = \left( {\alpha '_{s} E\left( {x_{i} } \right)} \right)^{{\gamma_{s} }}$$

where $\gamma_{s}$ is an exponent corresponding to study *s.* A value of $\gamma_{s}$ different from unity indicates a lack of proportionality between the mosquito sampling methods. In addition, $\alpha {\prime }_{s}$ will differ from $\alpha_{s}$ if $\gamma_{s}$ is different from unity.

The validity of the calibration was checked using published data sets from three sites on Bioko Island in Equatorial Guinea with an *An. gambiae* sensu stricto (s.s.) and *Anopheles melas* mixture [24] and for an urban site (Dar es Salaam in Tanzania) (99% *An. gambiae* s.s. and 1% *Anopheles arabiensis*) [15] by comparing with the credible intervals of models fitted to the pooled multi-study data resource described above.

## Results and discussion

**Figure 2:**
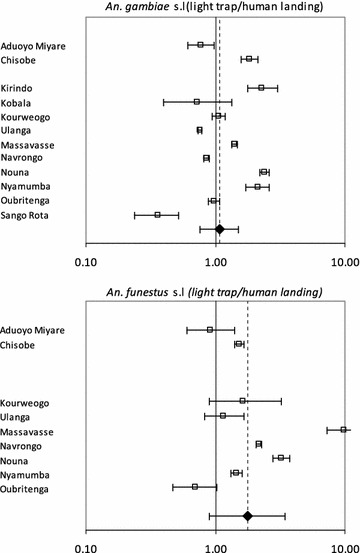
Study specific and overall estimates of sampling efficacy. Forest plots giving the estimated sampling efficacy of LT relative to HLC on a logarithmic scale, point estimates and 95% credible intervals of model (1) for *An. gambiae* s.l. (*top panel*) and *An. funestus* s.l. (*bottom panel*). The *dashed vertical lines* indicate the best estimates of the overall average sampling efficiencies.

The most abundant vector taxon sampled by either method across the sites and studies was *An. gambiae* sensu lato (s.l.) (Table 2). In the sites in Zambia (Chisoba and Nyamumba) *An. funestus* s.l. was more often sampled than *An. gambiae* s.l. For *An. gambiae* s.l., 12 studies were included, whereas for *An. funestus* s.l., only nine studies provided sufficient data. Forest plots (Figure 2) show point and interval estimates from the linear model of the relative sampling efficacy (Eq. 1) in each study, as well as the estimate of the overall average sampling efficacy. LT often collected greater numbers of mosquitoes than HLC (in seven out of 12 studies, LT caught more *An. gambiae* s.l. than HLC, and in seven out of nine studies, LT caught more *An. funestus* s.l. than HLC), although this relation varied between studies, even between sites adjacent to each other in Kenya or Zambia, and between sequential studies within a single site in Ulanga, Tanzania. In a comparison between the *An. gambiae* complex and the *An. funestus* group, the relative sampling efficacy of LT was greater for *An. funestus* s.l. than for *An. gambiae* s.l. for the studies in Navrongo and Ulanga 2004, but lesser for Nyamumba. For the other six studies where a comparison between species complexes was possible, the 95% credible intervals of the sampling efficacy estimates for these two vector taxa overlapped. The overall estimate of sampling efficacy of LT relative to HLC, estimated by pooling data from all studies and allowing for stochastic variability between studies, represented by the dashed vertical lines in Figure 2, was close to unity for both taxa [$\tilde{\alpha }_{t}$ = 1.06 (0.68–1.64) for *An. gambiae* s.l. and $\tilde{\alpha }_{t}$ = 1.37 (0.70–2.68) for *An. funestus* s.l.], meaning that on average it makes little difference whether LT collections are assumed equivalent to human biting rates, or if a conversion factor is used.

**Figure 3:**
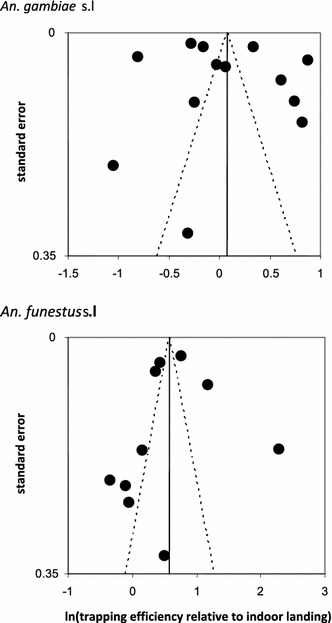
Representativeness of studied sites. Funnel plots giving the logarithm of the estimated sampling efficacy for each study (*horizontal axis*), standard error (se) of this estimate (*vertical axis*). The *vertical line* corresponds to the estimated overall average sampling efficacy. The *dashed lines* correspond to 95% pseudo-confidence limits calculated as 1.96 ± se within which 95% of the points are expected to occur in the event that the differences between studies arise only because of sampling variation.

Funnel plots (Figure 3) [39] were used to examine evidence for selection bias in these results. The points in these plots are expected to form a triangular pattern centred on the best estimate of the average sampling efficacy, which corresponds to the vertical line. An asymmetric funnel would indicate a relationship between treatment effect and study size, suggesting either a selection bias or a systematic difference between smaller and larger studies. In Figure 3, a large proportion of the points fall outside the dashed triangles, indicating that there was much more variation between studies in the estimated sampling efficacy than was expected if the true value of the efficacy was the same in each study. However, there is no indication of any systematic bias either upwards or downwards in the averages, since there are points scattered either side of the vertical lines, more or less independently of the standard error.

**Figure 4:**
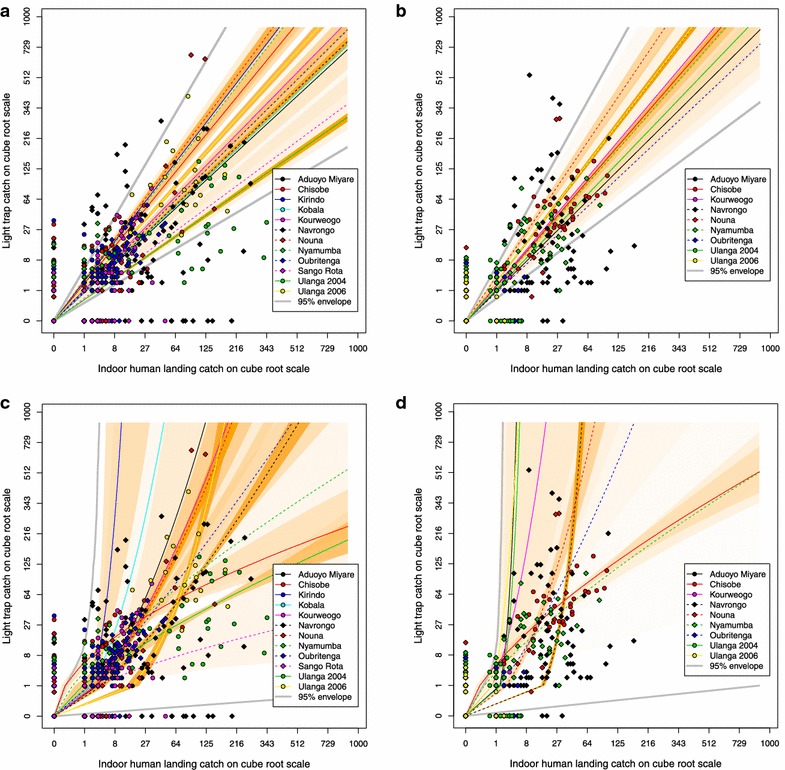
Fitted sampling efficiencies of light traps relative to human landing catches. *Each point* corresponds to a single matched set of mosquito collections; the *shaded orange polygons* correspond to 95% credible intervals for the best fitting curves, estimated for each site, with the transparency of the *orange colouring* inversely proportional to the surface area of the polygon. The *grey lines* demarcate the envelope within which 95% of fitted curves of unobserved studies are expected to fall based the variation observed in the studies used in this analysis (based on the joint posterior distributions of $\alpha_{s}$ and $\gamma_{s}$). Panel **a** shows the linear model for *An. gambiae*; panel **b** shows the linear model for *An. funestus*; panel **c** shows the power model for *An. gambiae*, and panel **d** shows the power model for *An. funestus.*

Figure 4 panels a and b and plots in Additional file 1 show the fitted linear relationships (Eq. 1) for each study, indicating how the variation shown in Figure 2, translates into different lines relating the numbers of mosquitoes caught by the two methods. Figure 4 panels c and d and plots in Additional file 1 show the fitted non-linear relationship (Eq. 2) between both methods in the numbers of mosquitoes collected. The comparisons between the lines in Figure 4a and the curves in Figure 4c indicate how much the relative sampling efficiencies for *An. gambiae* s.l. depend on mosquito density. Similarly, Figure 4b and d allow the corresponding comparisons for *An. funestus*. There is considerable variation among studies in the shapes of the curves in Figure 4c or d and few of the curves for the individual studies are close to being straight lines. For *An. gambiae* s.l., out of 12 studies included in the analysis, the only study in which the 95% credible interval estimate for $\gamma_{s}$ included the value of unity, so proportionality could not be excluded, was Nyamumba in Zambia. For eight of the 12 studies, the LT:HLC ratio increased as the number of mosquitoes increased (corresponding to $\gamma_{s} > 1$), and for three studies (Chisobe, Sango Rota and Ulanga 2004), it decreased (Table 2). Interestingly, this ratio increased with mosquito density in the Ulanga 2006 study, even though this study was done in the same location as the Ulanga 2004 study in which it decreased. Similarly, while this ratio increased with mosquito density at three of the studies in the same region of western Kenya (Aduoyo Miyare, Kirindo, Kobala), it decreased in the fourth (Sango Rota).

For *An. funestus* s.l., out of the nine studies included in the analysis, the 95% credible interval estimate for $\gamma_{s}$ included the value of unity for only three studies (Kourweogo, Oubritenga and Ulanga 2006) indicating that proportionality could not be excluded for these studies (Table 2). In four other studies (Aduoyo Miyare, Navrongo, Nouna and Ulanga 2004), the relative sampling efficacy of LT increased with increasing mosquito density (i.e., the slope in Figure 4 increased as the density increased). In the two remaining studies (Chisobe and Nyamumba), the slope decreased as mosquito density increased. No consistent geographical pattern was observed in the relative sampling efficacy of LT in sampling either species. In addition to the contrasting results for *An. gambiae* s.l. of the two sequential studies at the same village in Ulanga, the relationship varied also strongly among studies in geographically close sites, such as those located in neighbouring districts of western Kenya and Burkina Faso. Where $\gamma_{s}$ was significantly different from unity for both species complexes, this was in the same direction for both species ($\gamma_{s} > 1$ for Aduoyo Miyare, Navrongo and Nouna, and $\gamma_{s} < 1$ for Chisobe).

Despite the very limited precision of LT and inconsistent relationship with HLC, they may nevertheless be useful for comparing vector densities across different locations and time periods: mean catch per trap per night with LT was closely correlated to the mean catch per person per night by HLC for both *An. gambiae* s.l. and *An. funestus* s.l. The potential utility of the hierarchical models, assuming either simple linear proportionality or density-dependent relative sensitivity curves, was therefore explored to determine how best this multi-study dataset of unprecedented size might be exploited to generate approximate but broadly applicable calibration functions, for LT catches compared with HLC, with which to estimate human indoor exposure rates to malaria mosquitoes.

The hierarchical models allowing for deviations from proportionality clearly fit better than the simpler linear relationships, with the deviance information criterion (DIC) being much lower for the non-linear model (Table 3). While the Bayesian approach does not formally provide *p* values, these DIC values indicate that the improved fit of the power model cannot be attributed to chance alone. However, the variation in shapes of the multiple fitted curves within this hierarchical model (Figure 4; Table 2) result in very wide credibility intervals. After comparison of the results of these multi-study hierarchical models with the Bioko and Dar es Salaam data sets, it is clear that while the credibility intervals of the simple linear models are very wide, they are nevertheless precise enough to distinguish one vector population that responds to LT in a typical fashion and three that do not. The data from *An. melas* in Arena Blanca (Luba), Bioko, fit within the credible interval of the linear hierarchical model and are, therefore, very approximately consistent with the levels of relative trapping efficacy observed in these 13 studies from other rural areas (Additional file 2). However, in the two other sites on Bioko, Mongola and Riaba, and also in urban Dar es Salaam, where relative sensitivity of LT was so poor that it forced local surveillance teams to develop their own tent trap [15–17, 40], all the data lie below the lower boundary of the credibility interval for the linear model (Figures 2.2–2.4 in Additional file 2). In Mongola, Riaba and Dar es Salaam, LT are inadequate as a method for measuring host-seeking anopheline densities, unlike in the 13 rural studies to which the models were fitted. The site in Mongola is lit up at night due to a lit road and an airport, and also urban Dar es Salaam is lit up. However, Riaba is a rural site so night lights do not appear to be a likely explanation for the poor performance of LT at this site.

**Table 3:** Summary of fit of statistical models

Model	Deviance (ergodic average) $\bar{D}$	Effective number of parameters ($p_{D}$)	Deviance information criterion (DIC) [75] $DIC = p_{D} + \bar{D}$
Model 1	26,579.7	854.4	27,434.2
Model 2	22,656.8	867.9	23,524.6

The model with the lower DIC (Model 2) is the best fitting.

Even though the simple linear hierarchical model fit is also imprecise, the linear model can be used to compare to calibration data from other study sites, to determine whether LT catch malaria vector mosquitoes with sufficient efficacy to be useful and to very approximately relate these to biting rates experienced by exposed humans sleeping indoors. An R script is provided with an example (Additional files 3, 4) to allow other investigators to determine whether their locally-relevant calibration data are consistent with the observation of very approximate simple mean equivalence across these 13 geographically diverse studies.

As with other comparisons of LT with HLC in Africa [8, 9, 24], these results lead to a conclusion that the numbers of mosquitoes caught in LT placed beside human-occupied bed nets are only a very approximate indicators of the rates at which unprotected humans are exposed to mosquito bites. There were wide variations in the relative sampling efficiencies of LT and HLC, both across studies, even if they were very close to each other, and within study sites as time passes. While imprecision of the observations made with either method are to be expected based on the strong aggregation or overdispersion that is typical of mosquito catch data [35, 41–43], the matched experimental designs applied in each of these studies should yield data that are unbiased with respect to sampled times, locations and houses, natural variations in attractiveness [44–46] and technical skills of individual of human volunteers within studies, providing the relative sampling efficacy remains constant.

With both methods, it is challenging to trap mosquitoes in truly representative locations. The original procedures for HLC recognized the need to allow for differences in location between mosquito collectors and the general human population [47] (in particular in terms of whether they are inside or outside houses), but studies that adjust estimates of exposure for the use of interventions (such as repellents, house-screening and bed nets) [48, 49] have been the exception. Although it remains the standard, there is no obvious reason why HLC should be more reproducible than LT, especially as they are not automated and rely heavily on manual execution by volunteers working at times of the night when human performance of any active task is least effective [50, 51]. The substantial variations between studies in relative trapping efficacy are therefore unsurprising given the fundamentally crude nature of both techniques as applied in real-world situations [4, 6] and the difficulties in standardizing them [52]. Subtle differences in procedures could therefore explain some of the variation between studies.

Different *Anopheles* species are known to respond differently to LT. Studies of Cuban [25], Brazilian [26], Papua New Guinean (PNG) [27, 53] and Venezuelan [28] vectors have found LT to catch far fewer *Anopheles* than HLC, while with other vectors (in particular in a study of *Anopheles fluviatilis* in India [29] and another study of Malaysian vectors [30]) relative trapping efficacy has proven highly variable. In general, LT have been used mainly to capture African malaria vectors for which they appear to be relatively reliable, but a single mosquito taxon may react to LT very differently in nearby locations. In coastal Tanzania, LT appear to work well in all rural areas [10, 54] (Figure 4) but not in Dar es Salaam [15, 16], even though the most common malaria vector in all these studies are predominantly *An. gambiae* s.s., for which no geographic gene flow barriers are apparent along the mainland coast [55]. This suggests that temporal and geographic variations in the general environment and the dynamic mosquito populations it supports differentially affects the performance of the different methods, and/or that that there are subtle variations within the same taxon in terms of the innate mosquito characteristics that affect relative trapping efficacy. Most method comparison studies do not distinguish between species within the *An. gambiae* s.l. complex or the *An. funestus* s.l. group, even though they may well behave quite differently when they encounter either a LT beside an occupied bed net or an alert human sitting on a stool with a torch and aspirator. Sibling species have been reported as differentially sampled by the two techniques [15] and there may well also be relevant genetic variations within sibling species. For instance, LT may be biased towards sampling mosquitoes that are infected with malaria sporozoites [9, 10, 27], although infection rates are too low for this to explain most of the variation observed between studies here. The curvilinear relationships of relative trapping efficacy with density imply that some factor(s) affecting trapping efficacy vary with density within a mosquito population, although it is not known to what extent the sub-species composition changed over the seasons. This conclusion is reinforced by the different results of the two different Ulanga studies in the same place, separated only by a short period and using essentially identical methodologies. Given the dynamic nature of mosquito populations, and the demographic inevitability that peak mosquito densities are associated with recent emergences and relatively young populations, while low densities are often associated with aging populations [56], it may well be that much of the highly variable density-dependence observed here results from differential responsiveness of mosquitoes of different ages and/or physiological status.

Fortunately, it is not necessary to measure mosquito biting densities or EIR with any great degree of precision and even a very approximate estimate can be remarkably informative as an explanatory variable in epidemiological studies. Observed annual mean values for these indices of human exposure to mosquito bites and sporozoite inoculation exposure, respectively, vary spectacularly across logarithmic scales, spanning approximately four orders of magnitude [2, 57, 58]. Compared with this enormous range of variation in mean true values, even the variations in trapping efficacy described above are relatively small, so for most practical purposes it is reasonable to assume that LT catches are proportional, and approximately equivalent to the exposure of unprotected humans sleeping indoors. Over much of the range of transmission intensities of malaria in Africa there is saturation of prevalence in human populations so epidemiologically relevant measures of malaria prevalence, incidence, mortality and transmission stability are relatively insensitive to small changes in exposure. At measurable levels of EIR, large changes in transmission are required to achieve substantial changes in epidemiologically relevant indicators of malaria morbidity, mortality and parasite populations [2, 57, 58], so even the very loose approximation to proportionality suggested in Figure 4 is probably satisfactory for estimating transmission exposure in the vast majority of cases. So when HBR are estimated from LT catches, it is unlikely to make much practical difference how any conversion factor may be selected.

Malaria control and elimination require an indicator of malaria transmission exposure with sufficient precision to select appropriate intervention efforts. Considering the scales over which variations in the true mean EIR and the entomological measurement precision occur, the indicator on the entomological scale does not need not be very precise. The problem is analogous to selecting a rifle for hunting on the basis of the anticipated size of game animal pursued. A rifle selected to shoot a duck will suffice for any duck, regardless of whether it is a large or small one, spanning a three- to four-fold range of body mass. Similarly, a gun selected to shoot an impala, buffalo or elephant will suffice for any animal within the natural size range of that species. However, it is literally vitally important to distinguish between these four different target species which differ in size by approximately an order of magnitude. LT may, therefore, be very useful for measuring approximate biting or inoculation rates, and also inter-annual and spatial variation in these. Where available, they also provide better measures of seasonality in transmission than do indices measured in humans such as prevalence (where the oscillations are dampened owning to persistence of infection), or clinical incidence (where seasonality also depends on acquired immunity). The approximate proportionality in LT and HLC on average suggests that seasonal patterns estimated by different entomological approaches are likely to concur.

LT have proven invaluable for measuring malaria transmission in a wide diversity of contexts. Most of the EIR estimates used to support modern quantitative understanding of malaria were measured with LT and paint a remarkably consistent picture despite their limited precision [2, 57–59]. LT provide a low-cost way of achieving high sampling intensities for malaria vectors, most recently in longitudinal, community-based trapping schemes using solar-powered battery chargers [60]. They have been used many times to map vector-biting densities on very fine spatial scales [35, 41–43, 61–63]. Exposures estimated from LT data have been related with epidemiological outcomes in various settings in Africa [21, 60, 64–68] and provide valuable entomological measures of the impact of vector control on human exposure [69, 70].

## Conclusions

Overall, LT are an imprecise tool for measuring human exposure to indoor-biting African malaria vectors: variability between studies appears so important that no single effort to calibrate LT against HLC can be considered reliable: even where a single research team conducted sequential studies at the same site (Ulanga, Tanzania and both Zambian sites) or parallel studies at several neighbouring locations (all Zambian and Kenyan study sites), none of these studies yielded results that were consistent with each other. However, it is equally true to say that no consistent difference between the trapping efficacy of these two methods for estimating human exposure could be demonstrated, nor could any consistent relationship between relative trapping efficacy and vector biting density be demonstrated. The most practical and parsimonious approach to relating indoor LT catches to indoor human exposure appears to be to simply consider them as very approximately proportional and equivalent.

Local calibration exercises could be used to validate or invalidate the assumption of proportionality and equivalence by comparing the locally estimated mean sampling efficacy with the credibility intervals of the hierarchical simple linear model fitted to data from these 13 calibration studies carried out in rural areas distributed across Africa. If the local calibration estimate is below the lower limit of these multi-study credibility intervals, as was the case in one outlier study from an urban setting, LT most probably lack sufficient sensitivity for routine use in that context and alternative trapping methods should be evaluated. However, given that only large variations in transmission exposure have appreciable epidemiological relevance, and exposure may vary over four orders of magnitude in endemic areas of Africa, even such imprecise, approximate entomological estimates of biting or inoculation rates may nevertheless be useful. The clear track record of LT documented in the literature confirms that they remain an invaluable tool for studying malaria vector ecology and transmission epidemiology, so long as the substantial inconsistencies in trapping efficacy they exhibit are carefully considered in experimental design and data interpretation. Except where proven otherwise by comparison with the simple linear hierarchical model fit provided here (Additional files 2, 3, 4), LT may be considered a safe and approximately equivalent alternative to HLC for measuring indoor exposure to bites and malaria transmission by African vectors.

## Additional files

Additional file 1: Site specific plots. Additional file 2: Plots for additional data. Additional file 3: R script. Additional file 4: Sample data.
