# Supplementary material for: Applications and limitations of Centers for Disease Control and Prevention miniature light traps for measuring biting densities of African malaria vector populations: a pooled-analysis of 13 comparisons with human landing catches
Source: Malar J. 2015 Jun 18;14:247. doi: 10.1186/s12936-015-0761-9 (PMC4470360; doi:10.1186/s12936-015-0761-9)
Supplement: Additional file 1: — Site specific plots. [file 12936_2015_761_MOESM1_ESM.pdf]

Additional file 1

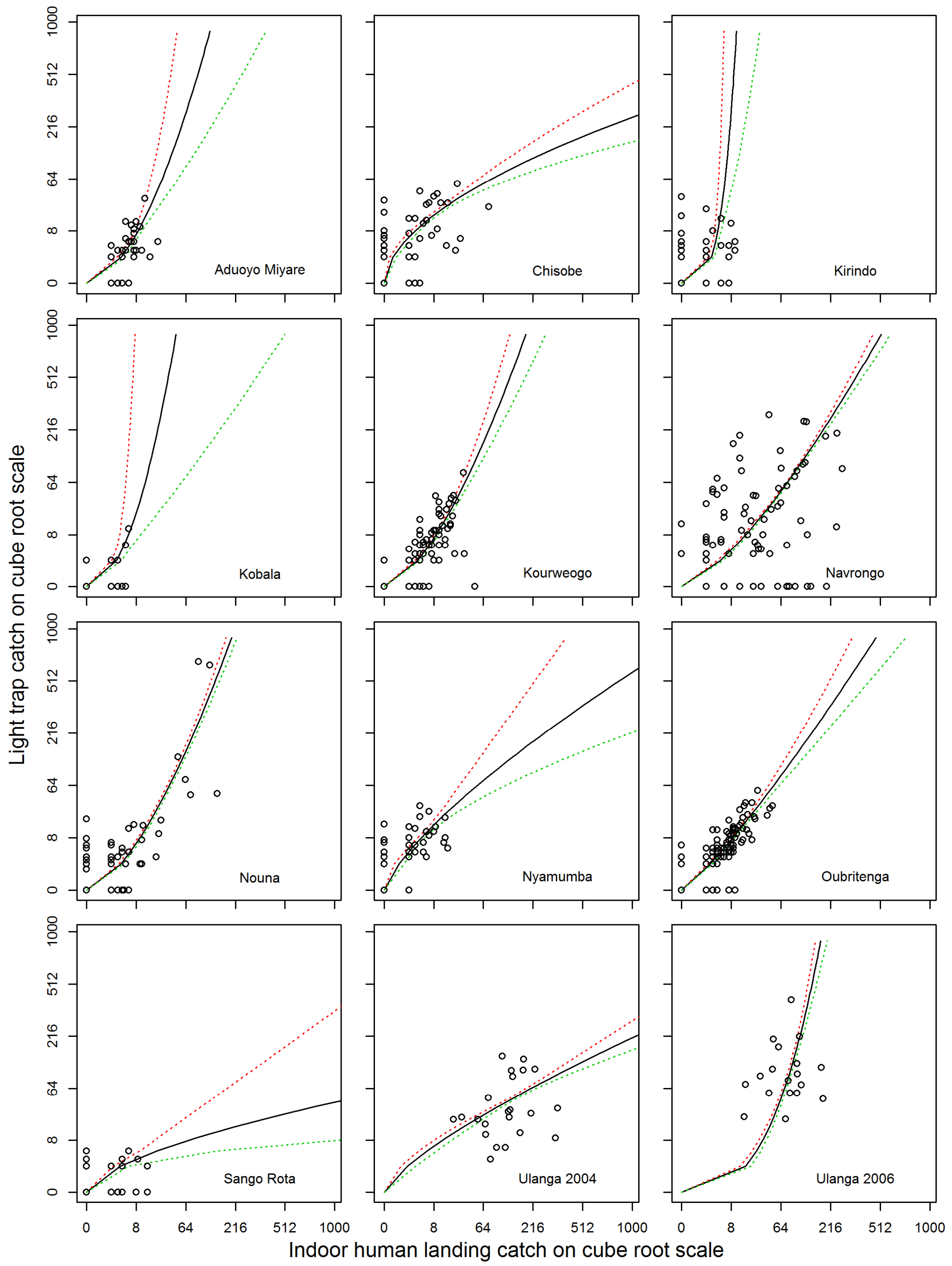

Figure A1. Site specific plots of the number of *An. gambiae* s.l. sampled by LT against those sampled by HLC, the straight line describes the relationship based on model 2.

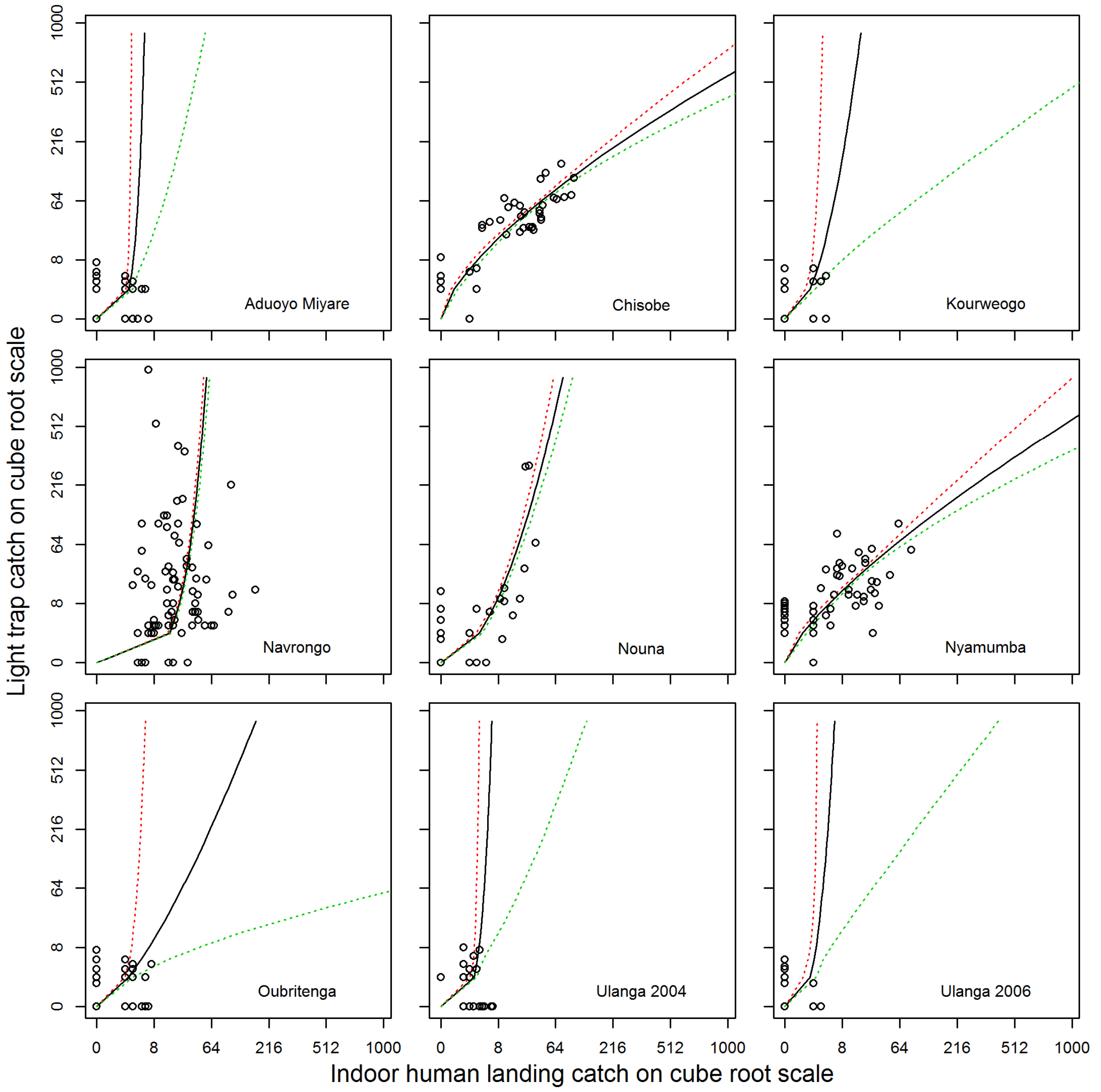

Figure A2. Site specific plots of the number of *An. funestus* s.l. sampled by LT against those sampled by HLC, the straight line describes the relationship based on model 2.
