# Supplementary material for: Applications and limitations of Centers for Disease Control and Prevention miniature light traps for measuring biting densities of African malaria vector populations: a pooled-analysis of 13 comparisons with human landing catches
Source: Malar J. 2015 Jun 18;14:247. doi: 10.1186/s12936-015-0761-9 (PMC4470360; doi:10.1186/s12936-015-0761-9)
Supplement: Additional file 2: — Plots for additional data. [file 12936_2015_761_MOESM2_ESM.pdf]

Additional file 2: Application to additional data

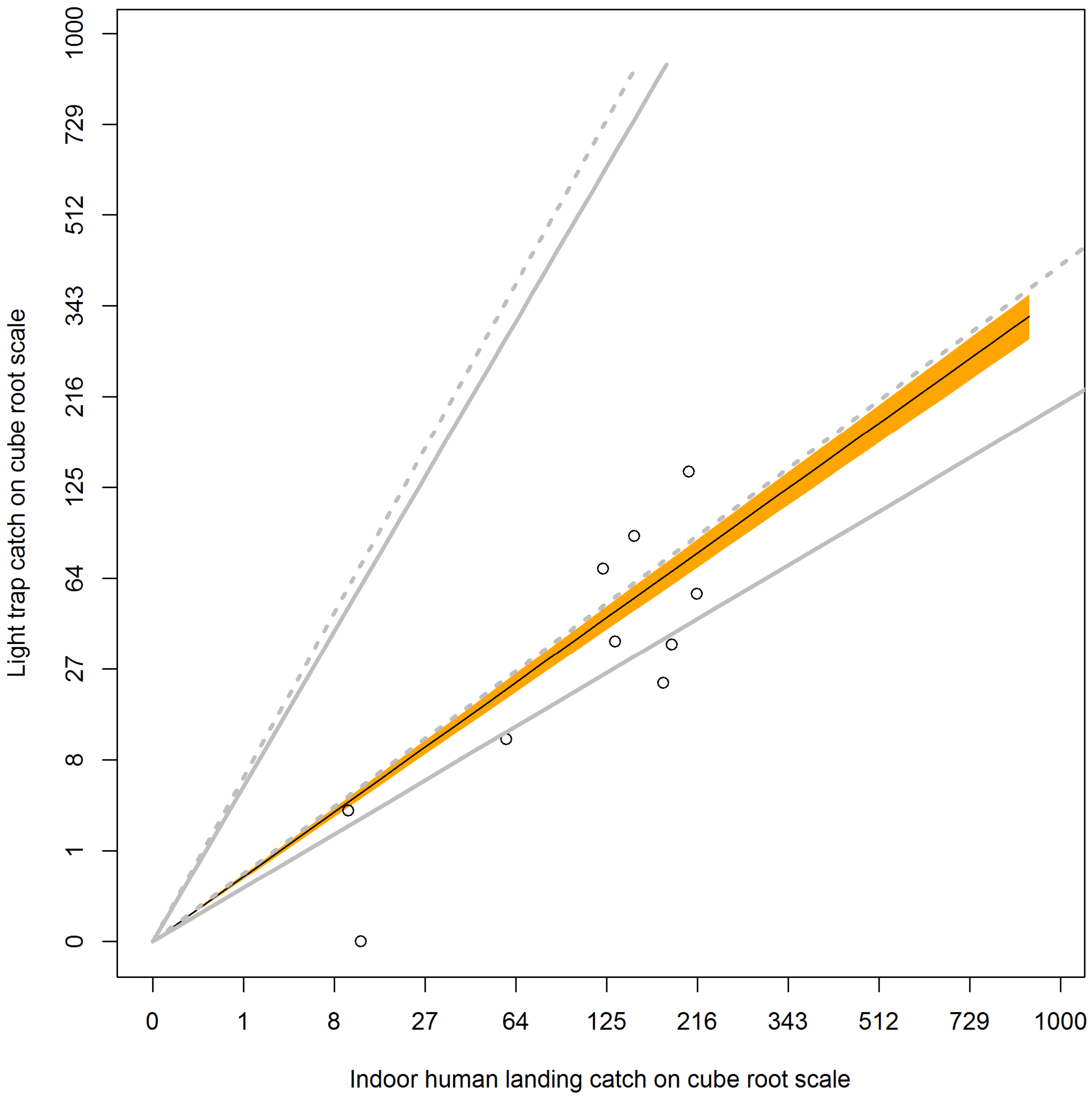

Figure 2.1. Relative performance of light traps to human landing catches of *Anopheles melas* in Arena Blanca (Luba), linear model. The black circles represent data from individual nights; the black line is the median of the fitted relationship; the orange polygon shows the 95% credible area for the mean of the relationship; the solid grey lines show the boundaries of the envelop within which 95% of fitted lines are expected to fall for *An. gambiae* s.l., and the dotted grey lines show the same for *An. funestus*, based on the analysis of relationships for 13 and eight sites, respectively. This figure was created by applying the R script (with a call to WinBUGS) in Additional file 3 to the data in Additional file 4. Data from Overgaard *et al.*, 2012 [http://www.malariajournal.com/content/11/1/56].

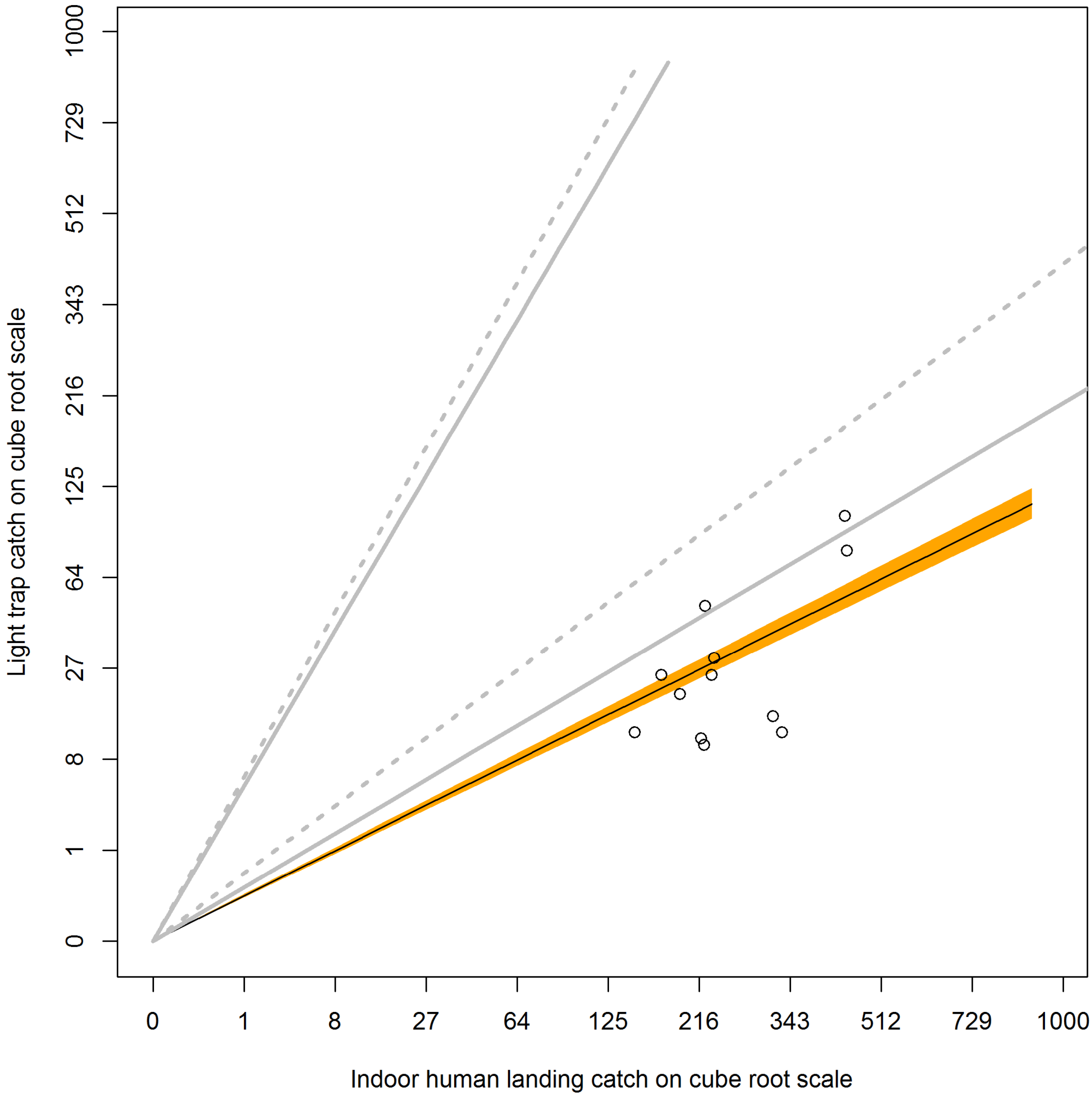

Figure 2.2. Relative performance of light traps to human landing catches of *Anopheles gambiae* s.s. in Mongola, linear model. Legend: see Figure 2.1.

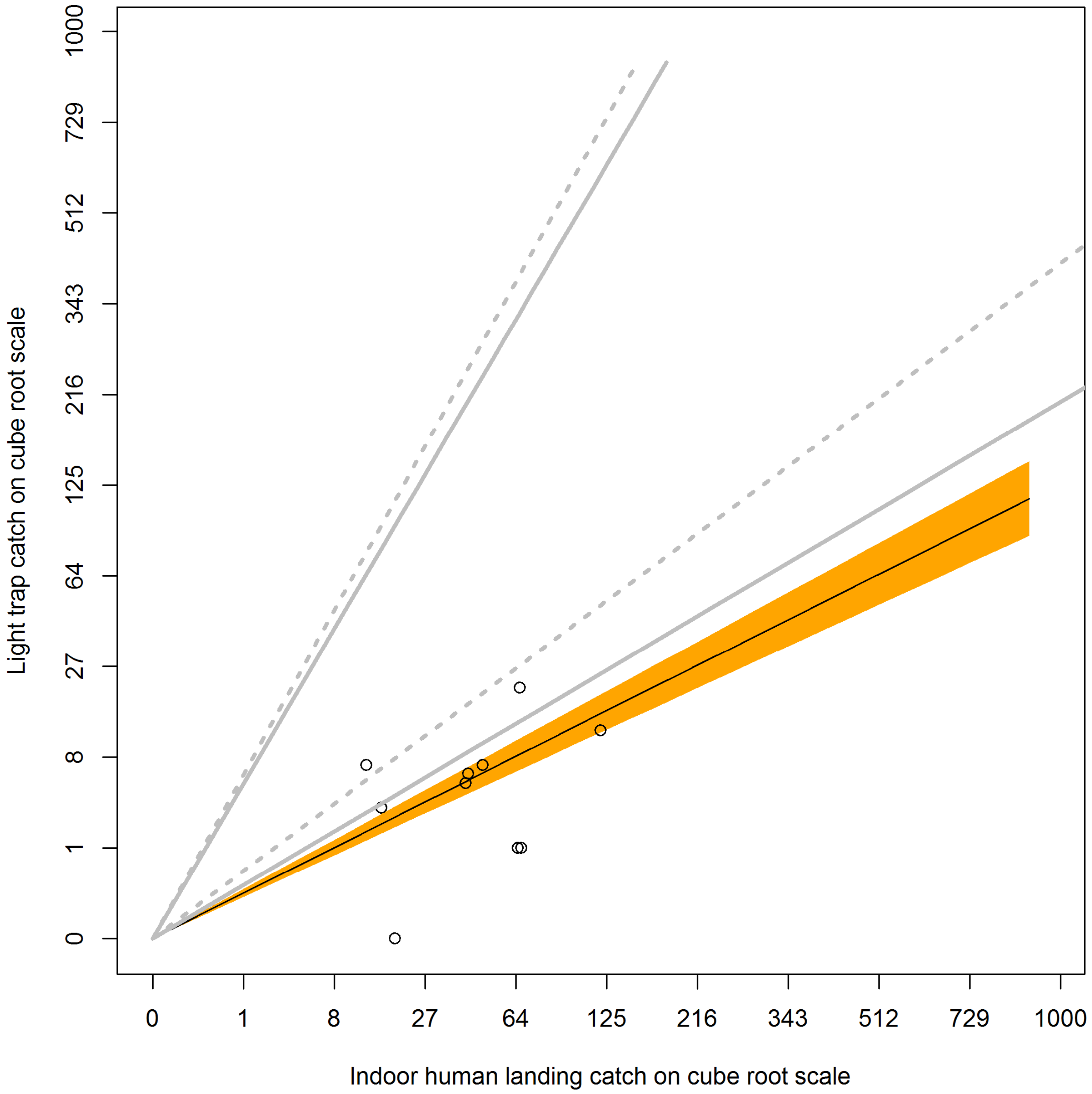

Figure 2.3. Relative performance of light traps to human landing catches of *Anopheles gambiae* s.s. and *Anopheles melas* in Riaba, linear model. Legend: see Figure 2.1.

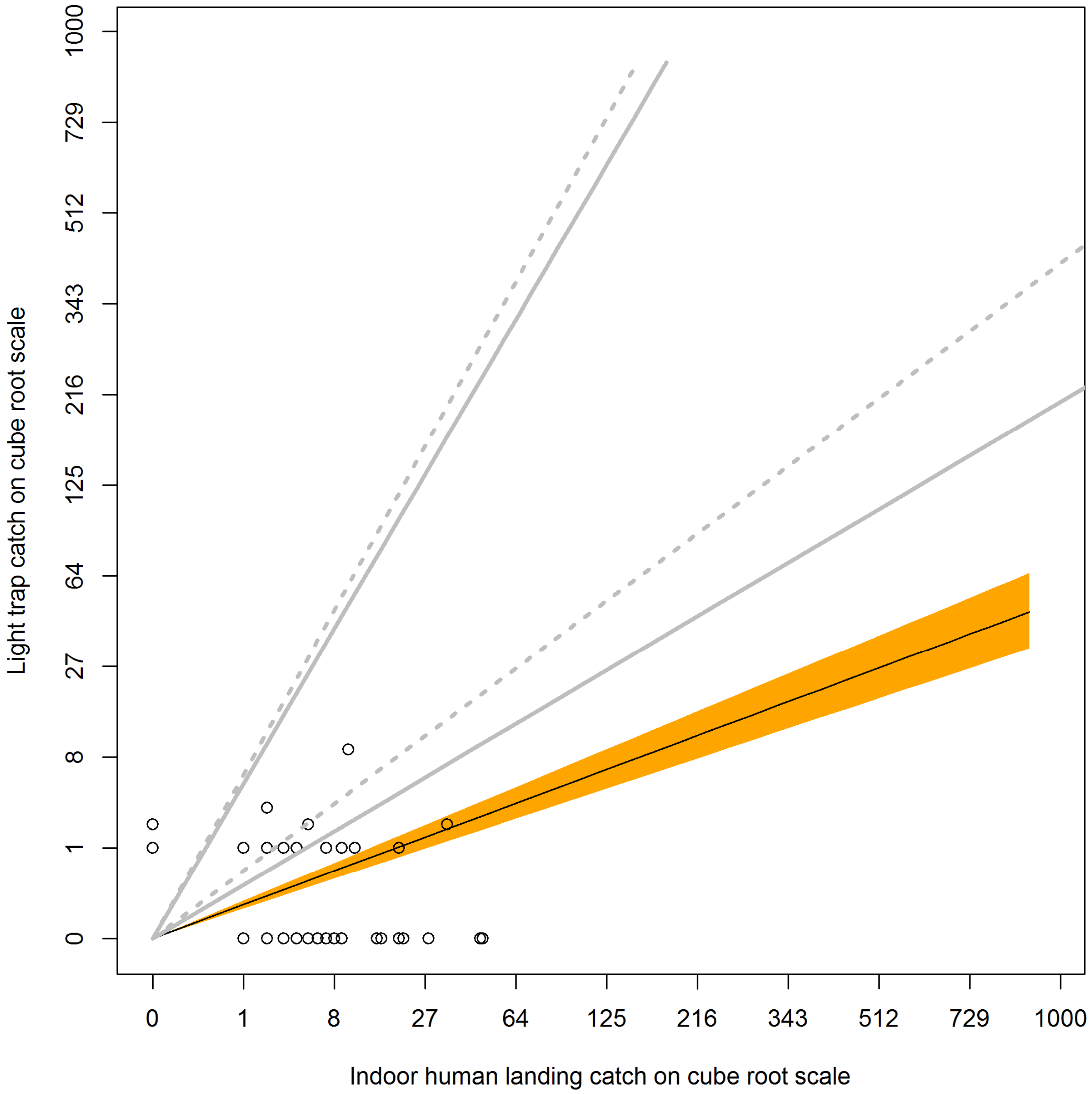

Figure 2.4. Relative performance of light traps to human landing catches of *An. gambiae* s.s. in Dar es Salaam, linear model. Legend: The black circles represent data from individual nights; the black line is the median of the fitted relationship; the orange polygon shows the 95% credible area for the mean of the relationship; the solid grey lines show the boundaries of the envelop within which 95% of fitted lines are expected to fall for *An. gambiae* s.l., and the dotted grey lines show the same for *An. funestus*, based on the analysis of relationships for 13 and eight sites, respectively. This figure was created by applying the R script (with a call to WinBUGS) in Additional file 3 to the data from Govella *et al.*, 2011 [http://www.parasitesandvectors.com/content/4/1/40]
